# Supplementary material for: A perivascular niche for multipotent progenitors in the fetal testis
Source: Nat Commun. 2018 Oct 30;9:4519. doi: 10.1038/s41467-018-06996-3 (PMC6207726; doi:10.1038/s41467-018-06996-3)
Supplement: Supplementary file 1 — Supplementary Information [file 41467_2018_6996_MOESM1_ESM.pdf]

Supplementary Information

A perivascular niche for multipotent progenitors in the fetal testis

Kumar and DeFalco

Supplementary Figures 1-9

Supplementary Tables 1-2

References

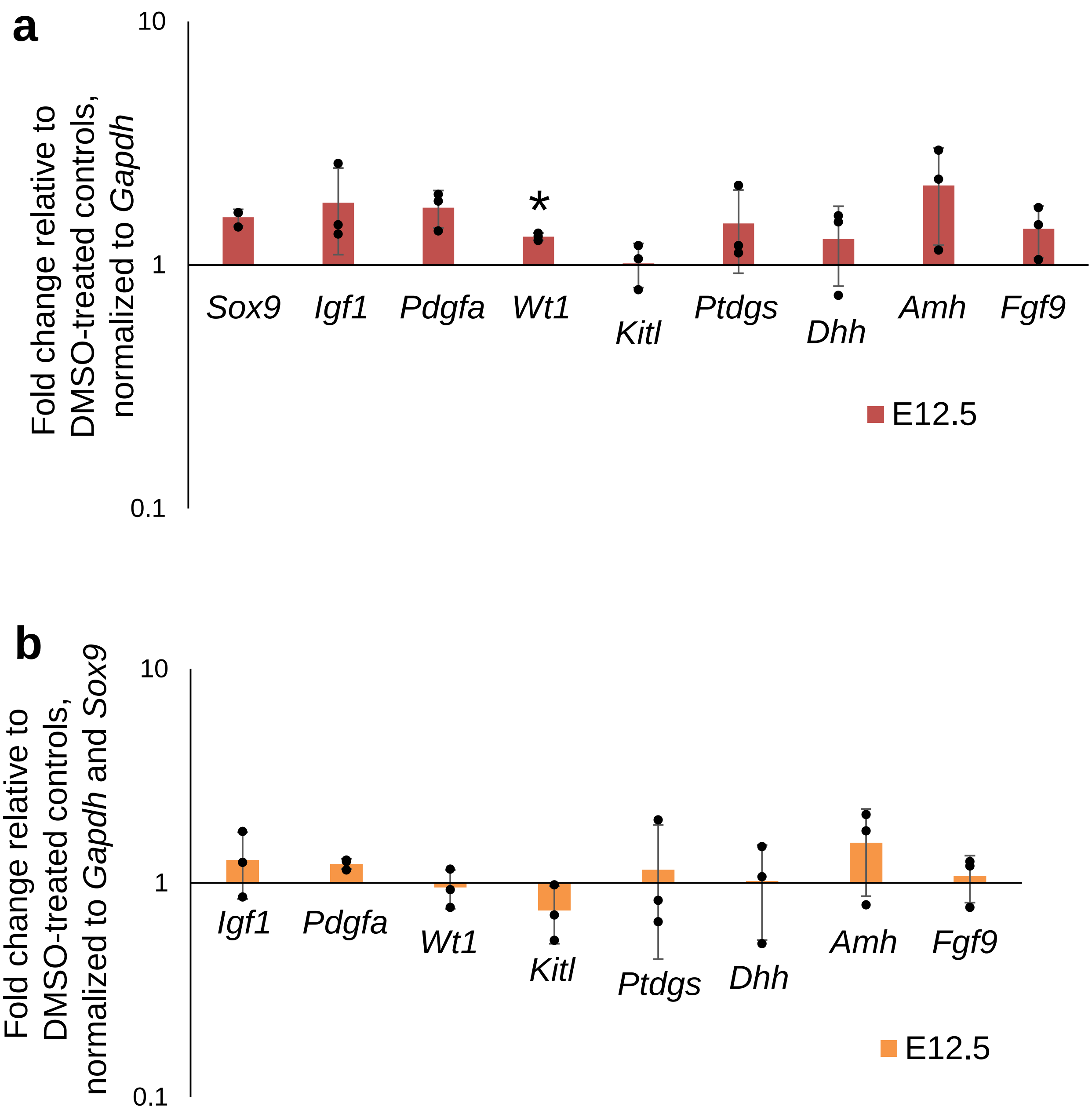

Supplementary Figure 1. Vascular disruption does not affect Sertoli cell differentiation or Sertoli-expressed factors involved in Leydig cell development. (a) qPCR analysis showing fold change in expression of Sertoli-cell-expressed factors involved in testicular differentiation and Leydig cell differentiation (*Sox9*, *Igf1*, *Pdgfa*, *Wt1*, *Kitl*, *Ptdgs*, *Dhh*, *Amh*, *Fgf9*) from whole E12.5 fetal testes cultured in the presence of VEGFR-TKI II for 48 hours relative to DMSO-treated controls. Samples were normalized to *Gapdh.* (b) qPCR analysis of the data in a, except additionally normalized to *Sox9* expression to account for changes in the relative contribution or proportion of Sertoli cells in the gonad in the absence of vasculature. All data are presented as the mean ± SD of three independent biological replicates (n=3 litters, ≥5 gonads/litter). *, $P < 0.05$ (two-tailed Student t-test).

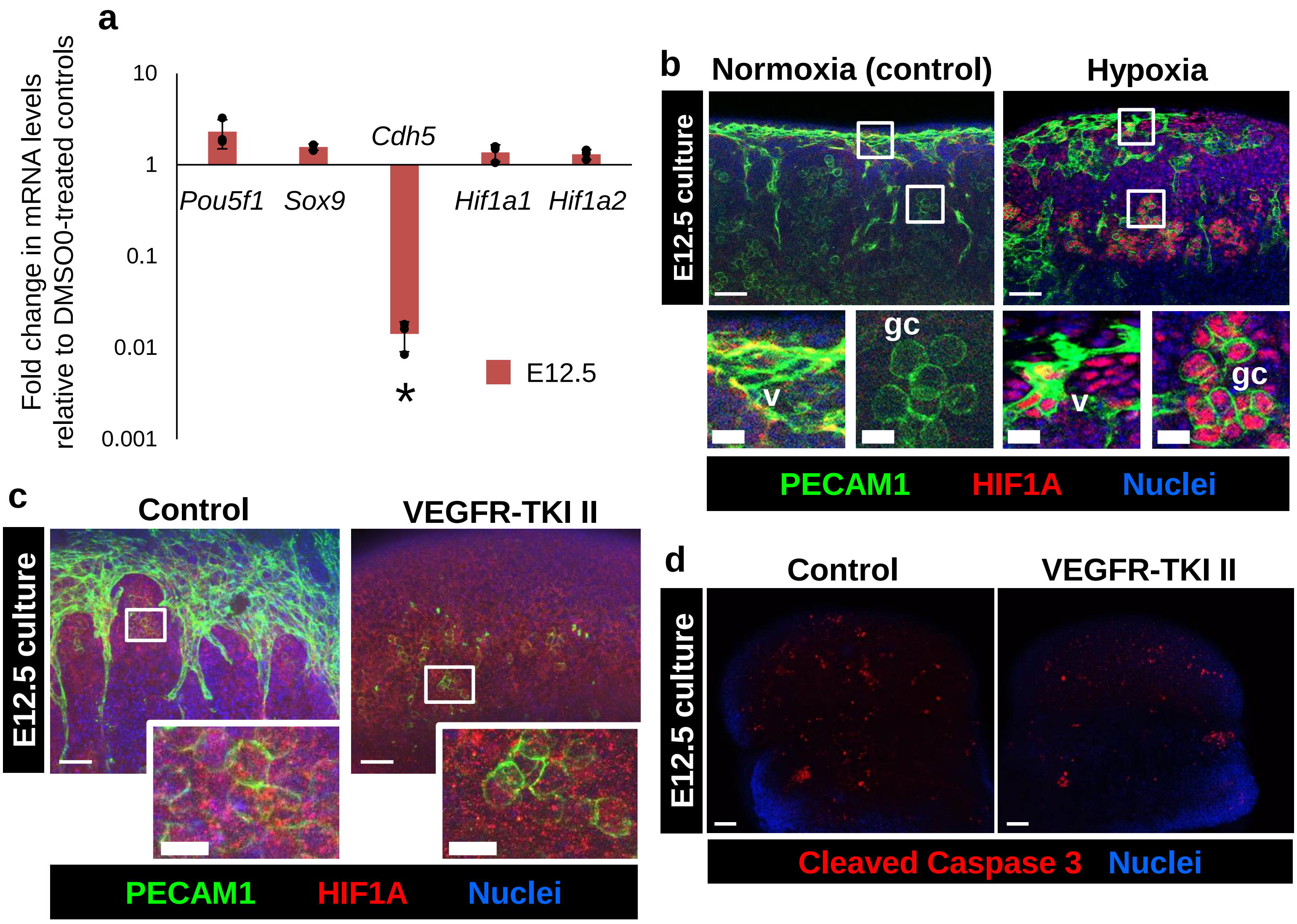

Supplementary Figure 2. Vascular disruption does not induce hypoxia or increase apoptosis in whole gonad explant cultures. (a) qPCR analysis showing fold change in expression of *Pou5f1* (marker for germ cells), *Sox9* (marker for Sertoli cells), *Cdh5* (marker for endothelial cells), and *Hif1a1* and *Hif1a2* (*hypoxia inducible factor alpha 1* isoforms 1 and 2; markers for hypoxia) in whole E12.5 fetal testes cultured in the presence of VEGFR-TKI II at normoxic conditions for 48 hours relative to DMSO-treated controls. Data are presented as the mean ± SD of three independent biological replicates (n=3 litters, ≥5 gonads/litter). *, $P < 0.05$ (two-tailed Student t-test). (b) Immunofluorescence images of E12.5 XY gonads cultured in normoxia (control) and hypoxia (1% oxygen) for 24 hours showing PECAM1 (marker for endothelial and germ cells) and HIF1A (marker for hypoxia) expression. Under hypoxic conditions (right) there is nuclear localization and stabilization of HIF1A in germ cells and vasculature compared to gonads cultured under normoxia (left); insets are higher-magnification images of the boxed regions focusing on vasculature (v) or germ cells (gc). (c) Images of E12.5 fetal testes cultured in the absence (Control, DMSO) or presence of VEGFR-TKI II for 48 hours. Insets are higher-magnification images of the boxed regions, showing similar weak expression and diffuse localization of HIF1A in germ cells in both conditions. (d) Images of E12.5 fetal testes cultured in the absence (Control, DMSO) or presence of VEGFR-TKI II for 48 hours, showing similar numbers of cleaved-Caspase-3-positive apoptotic cells under both conditions. Thin scale bars, 50 μm; thick scale bars, 10 μm.

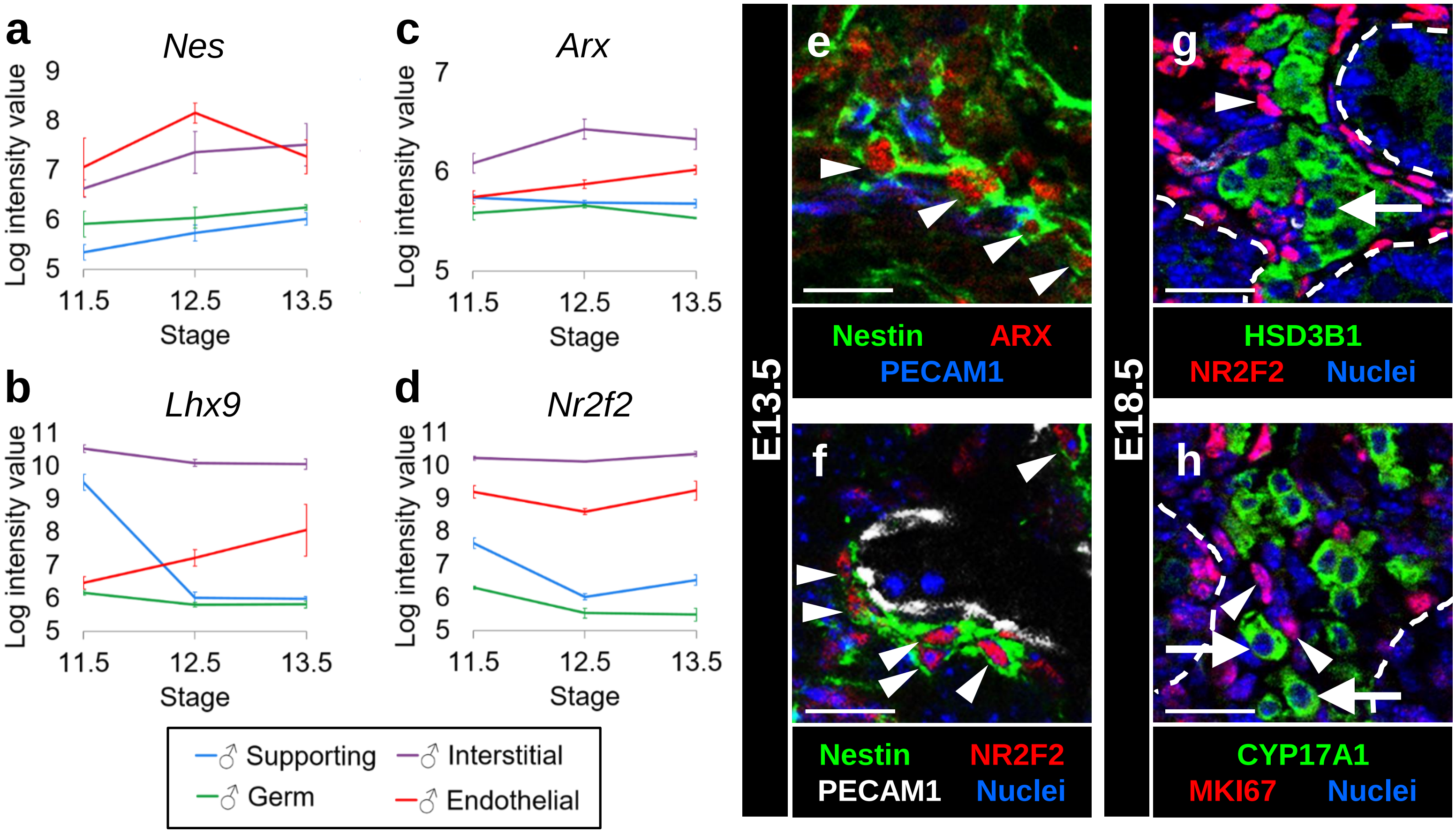

Supplementary Figure 3. Nestin-positive cells co-express markers of Leydig progenitors. (a-d) Plots showing gene expression of *Nestin* (*Nes*, a), *Lhx9* (b), *Arx* (c), and *Nr2f2* (d) generated from gonad lineage-specific microarray data[1], where cell lineages (supporting Sertoli cells, interstitial cells, endothelial cells, and germ cells) are independently plotted in different colors. Plot contains data for XY samples at stages E11.5, E12.5, and E13.5 for each cell type. In general, expression values below 6 are considered background levels of expression. For more information about this data set, see previous publication[1]. As we previously reported[1], our observation of *Nestin* expression in endothelial cells in our previous transcriptome analyses was likely due to aberrant interstitial expression of the *Kdr*-mCherry transgenic line used to isolate endothelial cells; well-characterized differentiated-Leydig-cell-specific genes such as *StAR*, *Cyp11a1, Cyp17a1,* and *Hsd3b1* also showed expression in endothelial cells in that study. (e-h) Immunofluorescence images of E13.5 (e,f) and E18.5 (g,h) wild-type CD-1 fetal testes. The nuclei of perivascular Nestin-positive cells are positive for ARX (e, arrowheads) and NR2F2 (f, arrowheads). In contrast to Leydig progenitors (g and h, arrowheads), differentiated Leydig cells (as marked by HSD3B1 or CYP17A1) do not express NR2F2 (g, arrow) or the active cell cycle marker MKI67 (h, arrows). Scale bars, 25 μm.

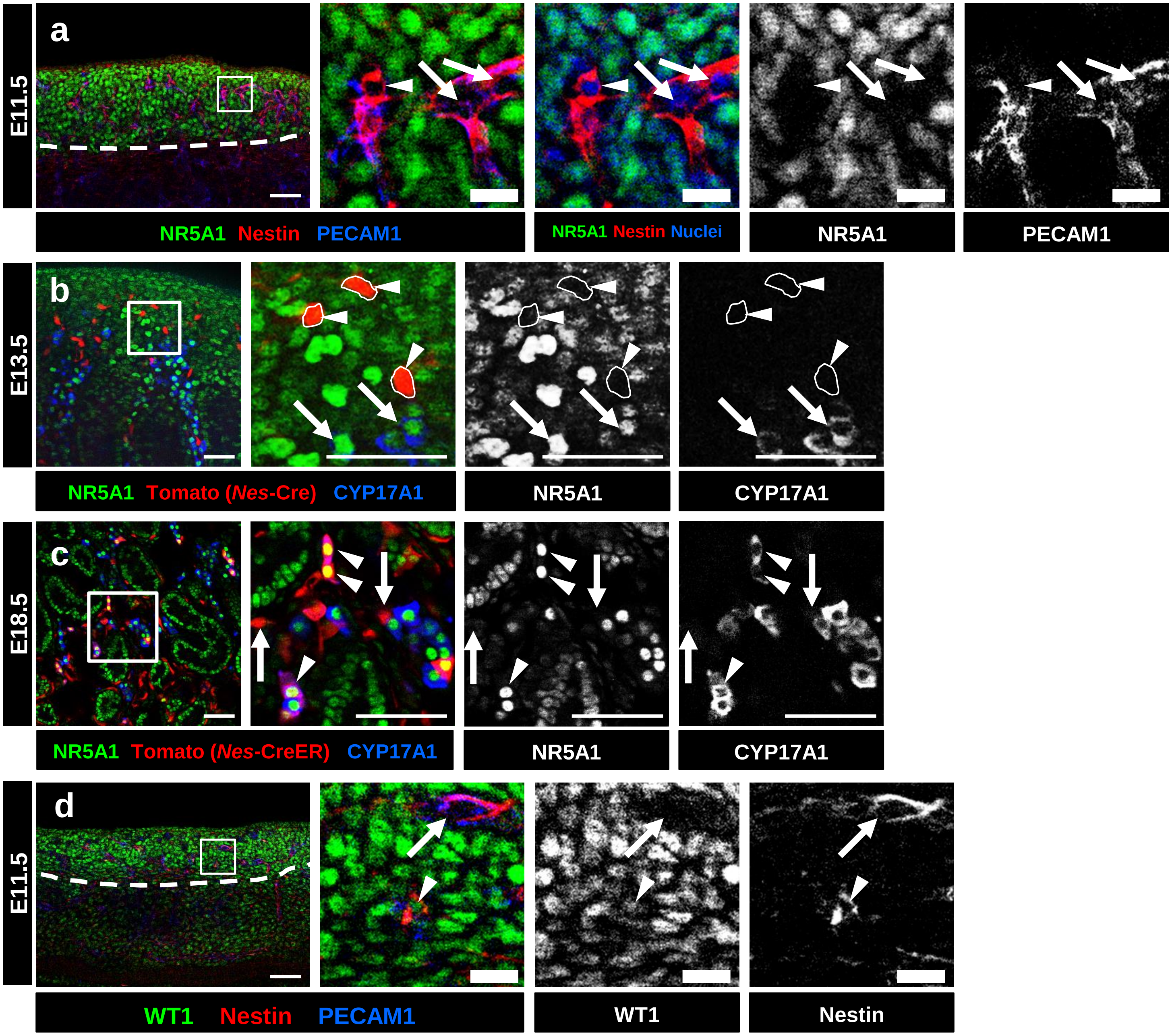

Supplementary Figure 4. Nestin-positive cells are WT1-positive but NR5A1-negative, consistent with a mesonephric origin. (a-d) Immunofluorescence images of fetal testes from E11.5 wild-type C57BL/6J (a,d), E13.5 *Nestin*-Cre;*Rosa*-Tomato (b), or E18.5 *Nestin*-CreER;*Rosa*-Tomato embryos exposed to 4-hydroxytamoxifen at E12.25 (c). Black-and-white panels on right show single-color channels as labeled. (a) Nestin-positive cell (a, arrowhead) is negative for NR5A1, similar to PECAM1-positive endothelial cells (a, arrows). (b) Tomato-labeled cells (white outlines and arrowheads) are NR5A1-negative, in contrast to CYP17A1-expressing fetal Leydig cells (b, arrows), which are NR5A1-positive like most other somatic cells in the fetal gonad. (c) By E18.5, Tomato-positive cells that differentiated into CYP17A1-positive fetal Leydig cells strongly express NR5A1 (c, arrowheads), in contrast to Tomato-positive undifferentiated interstitial cells (c, arrows), which remain NR5A1-negative. (d) E11.5 Nestin-positive cells (d, arrowhead) are positive for WT1, in contrast to endothelial cells (d, arrow). Thin scale bars, 50 μm; thick scale bars, 10 μm.

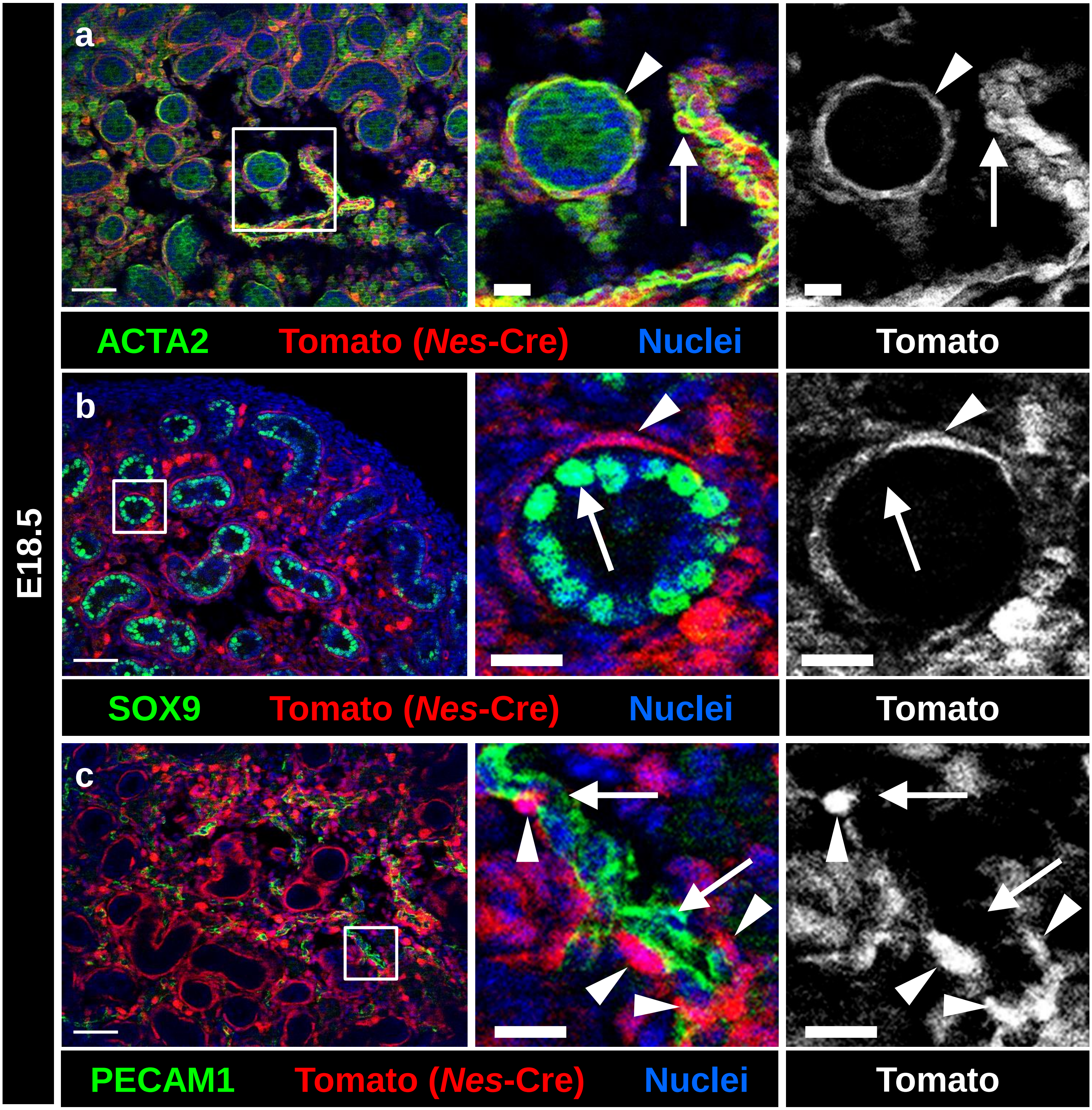

Supplementary Figure 5. Fetal *Nestin*-expressing cells give rise to peritubular myoid cells and vascular smooth muscle but not Sertoli or endothelial cells. (a-c) Immunofluorescence images of E18.5 *Nestin*-Cre; *Rosa*-Tomato fetal testes. Right-most panels show Tomato channel only. (a) Anti-ACTA2 (also called SMA; smooth muscle marker) staining shows that peritubular myoid cells around the testis cords (a, arrowhead) and vascular smooth muscle (a, arrow) are Tomato-positive. (b,c) Anti-SOX9 staining (b) and anti-PECAM1 staining (c) show that Sertoli cells (b, arrow) and endothelial cells (c, arrows) are Tomato-negative, while peritubular myoid cells (b, arrowhead) and interstitial perivascular cells (c, arrowheads) are Tomato-positive. Thin scale bars, 50 μm; thick scale bars, 10 μm.

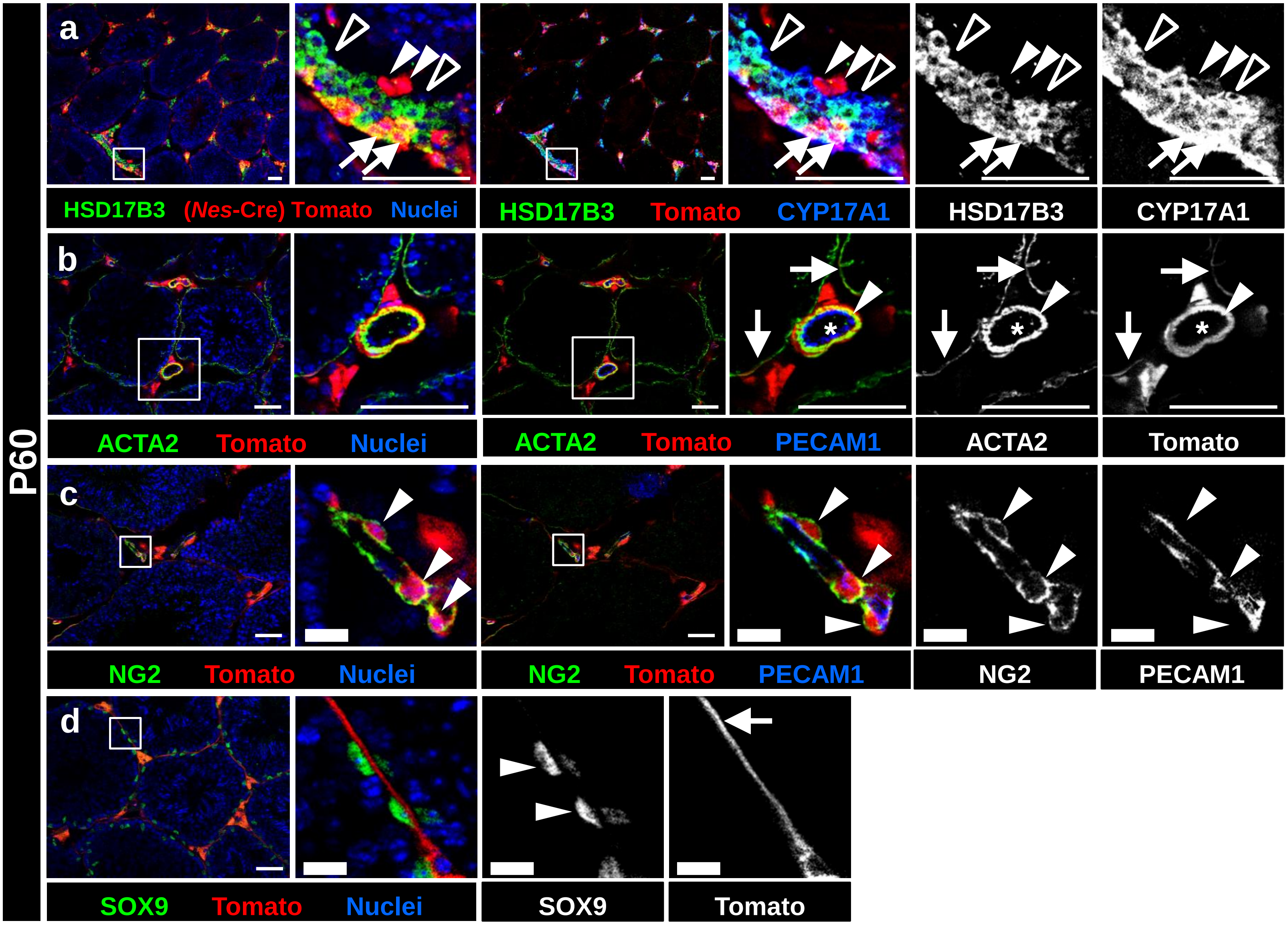

Supplementary Figure 6. *Nestin*-expressing cells give rise to adult Leydig cells (ALCs), vascular smooth muscle cells, peritubular myoid cells, and pericytes in the adult testis. (a-d) Immunofluorescence images of adult (P60) *Nestin*-Cre;*Rosa*-Tomato testes. Right-most panels show single channels as labeled. (a) Anti-HSD17B3 (an ALC-specific marker) staining shows that some ALCs (a, arrows) are Tomato-positive, as well as some retained FLCs (HSD17B3-negative; a, white arrowheads); some retained FLCs are Tomato-negative (a, black arrowheads). (b,c) Anti-ACTA2 (b) and anti NG2 (c) staining show that peritubular myoid cells around the testis cords (b, arrows), vascular smooth muscle (b, arrowhead), and pericytes (c, arrowheads) are Tomato-positive. PECAM1-positive endothelial cells within blood vessels (asterisk in b) are Tomato-negative. (d) Anti-SOX9 staining show that Sertoli cells (d, arrowheads) are Tomato-negative; arrow in d indicates Tomato-positive peritubular myoid cell layer. Thin scale bars, 50 μm; thick scale bars, 10 μm.

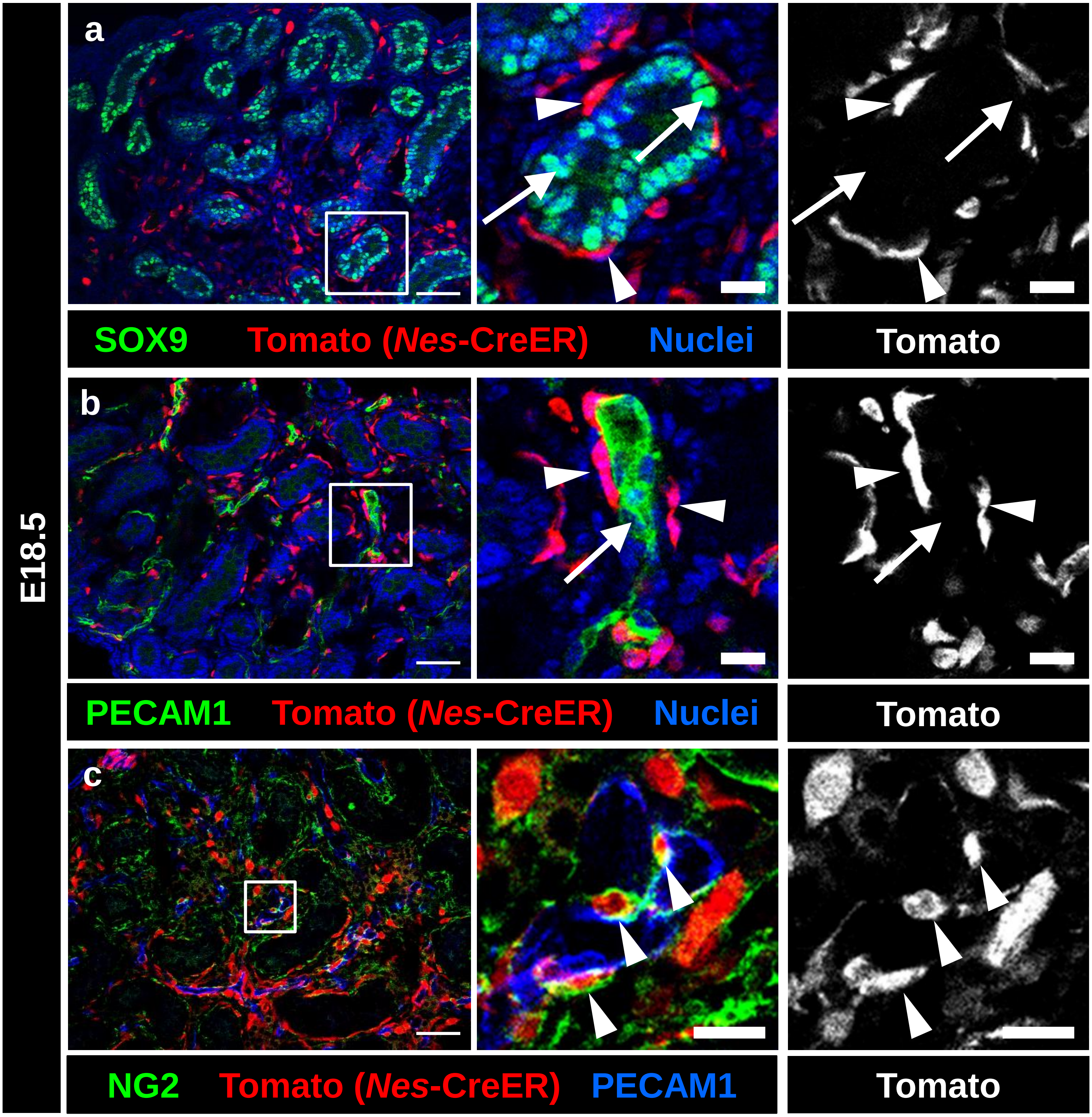

Supplementary Figure 7. Fetal *Nestin*-expressing perivascular cells are multipotent progenitors, but do not give rise to Sertoli or endothelial cells. (a-c) Immunofluorescence images of E18.5 *Nestin*-CreER; *Rosa*-Tomato testes from embryos exposed to 4-hydroxytamoxifen *in utero* at E12.25 to identify specific fates of initial perivascular *Nestin*-expressing cells in the fetal testis. Right-most panels show Tomato channel only. Anti-SOX9 staining (a) and anti-PECAM1 staining (b) show that Sertoli cells (a, arrows) and endothelial cells (b, arrow) are Tomato-negative, while peritubular myoid cells (a, arrowheads) and interstitial perivascular cells (b, arrowheads) are Tomato-positive. (c) Anti-NG2 staining (also called CSPG4; a marker for pericytes) reveals that pericytes (c, arrowheads) are Tomato-positive. Thin scale bars, 50 μm; thick scale bars, 10 μm.

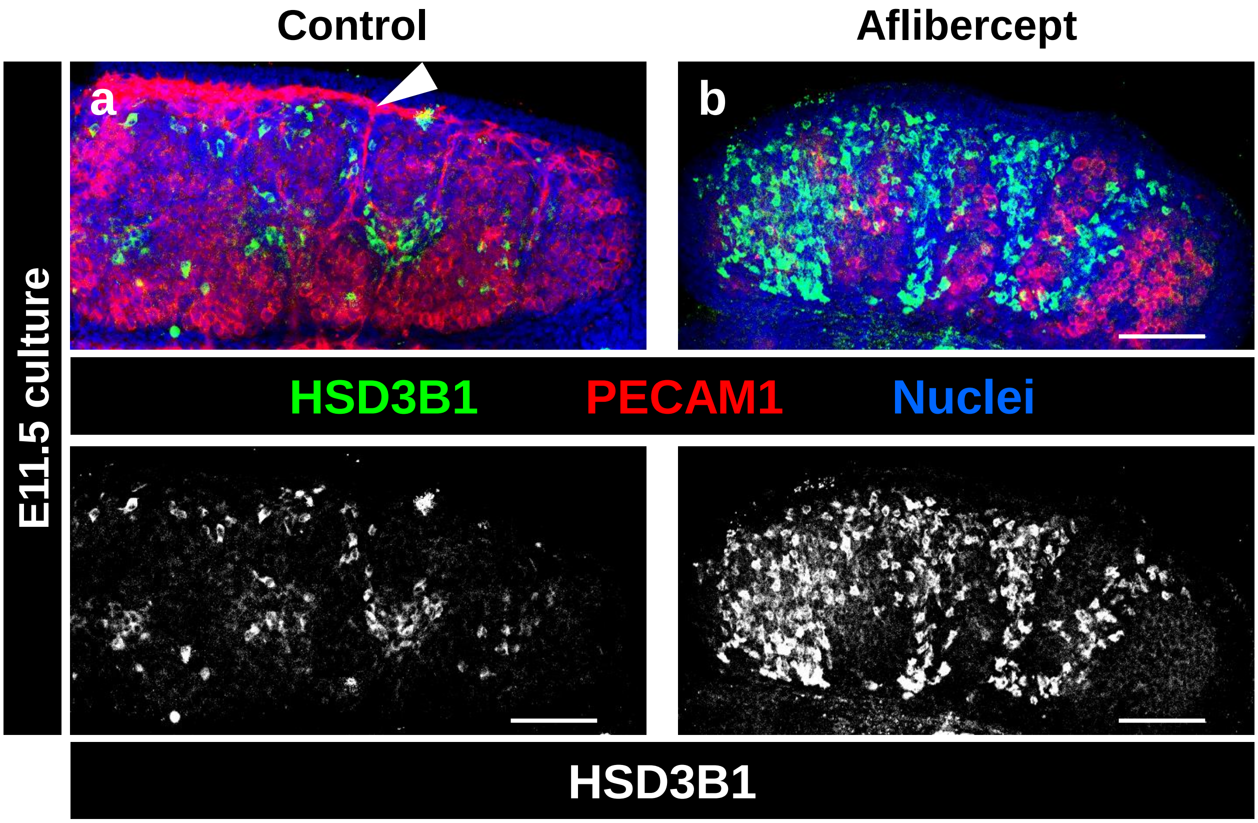

Supplementary Figure 8. A peptide-based, VEGF-specific vascular inhibition method leads to supernumerary Leydig cells. (a,b) E11.5 CD-1 testes were cultured for 48 hours after treatment with vehicle (a) or Aflibercept/VEGF-Trap (b). Aflibercept-treated testes show a lack of testicular vasculature (arrowhead in a) and an increased number of HSD3B1-positive Leydig cells. Scale bars, 100 μm.

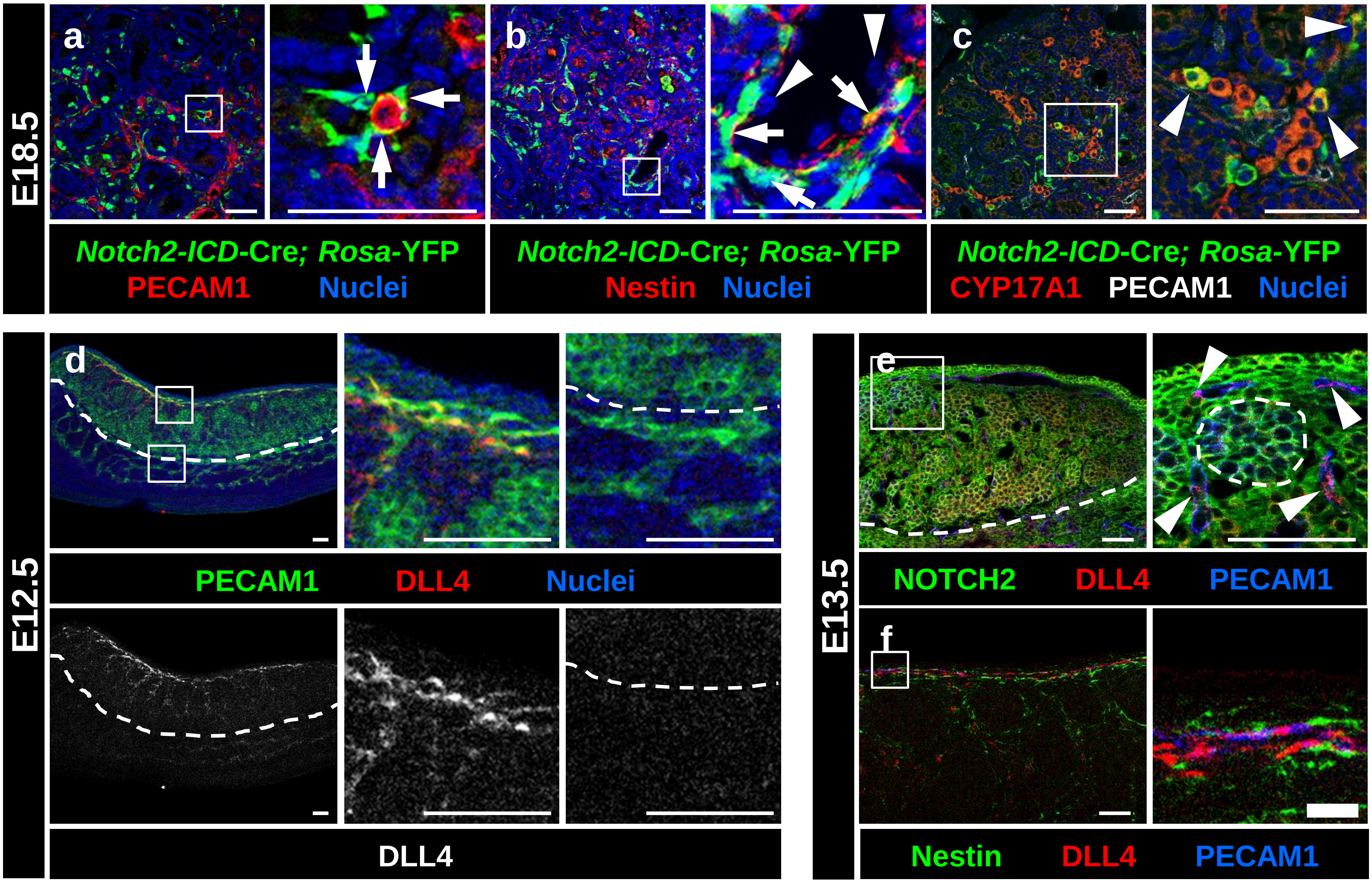

Supplementary Figure 9. DLL4 and NOTCH2 expression in the fetal testicular interstitium. (a-c) Immunofluorescence images of E18.5 *Notch2-ICD*-Cre; *Rosa*-YFP fetal testes. (d-f) Immunofluorescence images of E12.5 (d) and E13.5 (e,f) CD-1 wild-type fetal testes. (a,b) Perivascular Nestin-positive cells undergo active NOTCH2 signaling (YFP-positive; a and b, arrows), while endothelial cells are negative for YFP (b, arrowheads). (c) A subset of CYP17A1-positive FLCs are positive for YFP (c, arrowheads). (d) DLL4 is expressed only in endothelial cells (labeled with PECAM1). Black-and-white panels show single channels as labeled. (e) Anti-NOTCH2 staining shows that NOTCH2 is expressed throughout testis cords (dashed outline in e) and in the interstitial mesenchyme of the fetal testis, and next to DLL4-positive endothelial cells (e, arrowheads). (f) Anti-Nestin staining shows that DLL4-positive endothelial cells are in close proximity to Nestin-positive cells. Dashed outlines in d and e indicate gonad-mesonephros boundary. Thin scale bars, 50 μm; thick scale bar, 10 μm.

<u>Supplementary Table 1.</u> List of primary antibodies used for immunofluorescence**.**

**Antibody**	**Dilution**	**Source**
Rabbit anti-SOX9	1:4,000	EMD Millipore #AB5535
Rat anti-PECAM1	1:250	BD Biosciences #553370
Rat anti-CDH5	1:500	BD Biosciences #550548
Rabbit anti-Nestin	1:1,000	Biolegend #PRB-315C
Chicken anti-Nestin	1:200	Neuromics #CH23001
Goat anti-PECAM1	1:300	R&D Systems #AF3628
Goat anti-CYP17A1	1:500	Santa Cruz #sc-46081
Goat anti-HSD3B1	1:100	Santa Cruz #sc-30820
Rabbit anti-HSD3B1	1:500	Cosmo Bio #KAL-KO607
Rabbit anti-ACTA2	1:500	Abcam #ab5694
Rabbit anti-NG2	1:500	EMD Millipore #AB5320
Rabbit anti-MKI67 (Ki-67)	1:500	Thermo Fisher Scientific #RM-9106-S
Rat anti-phospho Histone H3 (Ser10)	1:500	EMD Millipore #MABE939
Rabbit anti-HIF1A	1:100	Abcam #ab179483
Rabbit anti-cleaved Caspase 3 (Asp175)	1:250	Cell Signaling #9661S
Chicken anti-GFP	1:1,000	Aves #GFP-1020
Rabbit anti-ARX	1:500	K. Morohashi; Kitamura *et al.*[2]
Mouse anti-NR2F2 (COUP-TFII)	1:500	Perseus Proteomics #PP-H7147-00
Rat anti-NR5A1 (SF1)	1:100	Cosmo Bio #KAL-KO610
Rabbit anti-RFP (tdTomato)	1:500	Rockland #600-401-379
Goat anti-DLL4	1:100	R&D Systems #AF1389
Rabbit anti-NOTCH2	1:1,000	Cell Signaling #5732S
Mouse anti-WT1 (F-6)	1:100	Santa Cruz #sc-7385
Rat anti-HSD17B3	1:200	Y. Shima; Shima *et al.*[3]

<u>Supplementary Table 2.</u> Sequences of primers used for quantitative real-time PCR (qPCR).

Gene name	Sequence (5’ to 3’)
Sox9 forward	GCGGAGCTCAGCAAGACTCTG
Sox9 reverse	ATCGGGGTGGTCTTTCTTGTG
Pou5f1 (Oct4) forward	GGAGGAAGCCGACAACAATGA
Pou5f1 (Oct4) reverse	TCCACCTCACACGGTTCTCAA
Cdh5 forward	TCCTCTGCATCCTCACTATCACA
Cdh5 reverse	GTAAGTGACCAACTGCTCGTGAAT
Nestin forward	GCTGGAACAGAGATTGGAAGG
Nestin reverse	CCAGGATCTGAGCGATCTGAC
Cyp11a1 forward	TGGCCCCATTTACAGGGAGAA
Cyp11a1 reverse	GGCATCTGAACTCTTAAACAGGA
Cyp17a1 forward	CAGAGAAGTGCTCGTGAAGAAG
Cyp17a1 reverse	AGGAGCTACTACTATCCGCAAA
Hes1 forward	ATAGCTCCCGGCATTCCAAG
Hes1 reverse	GCGCGGTATTTCCCCAACA
Hes5 forward	AGTCCCAAGGAGAAAAACCGA
Hes5 reverse	GCTGTGTTTCAGGTAGCTGAC
HeyL forward	CAGCCCTTCGCAGATGCAA
HeyL reverse	CCAATCGTCGCAATTCAGAAAG
Hey1 forward	GCGCGGACGAGAATGGAAA
Hey1 reverse	TCAGGTGATCCACAGTCATCTG
Hif1a1 forward	CGGCGAGAACGAGAAGAA
Hif1a1 reverse	CTTCGCCGAGATCTTGCT
Hif1a2 forward	CATCCATGTGACCATGAGGA
Hif1a2 reverse	CTCCGCTGTGTGTTTAGTTC
Amh forward	CCACACCTCTCTCCACTGGTA
Amh reverse	GGCACAAAGGTTCAGGGGG
Fgf9 forward	CAGGGAACCAGGAAAGACCA
Fgf9 reverse	GAGGTAGAGTCCACTGTCCAC
Pdgfa forward	CAGTGTCAAGGTGGCCAAAGT
Pdgfa reverse	TGGTCTGGGTTCAGGTTGGA
Ptgds forward	GCTCTTCGCATGCTGTGGAT
Ptgds reverse	GCCCCAGGAACTTGTCTTGTT
Wt1 forward	CGGTCCGACCATCTGAAGAC
Wt1 reverse	GTTGTGATGGCGGACCAATT
Kitl forward	TCTGCGGGAATCCTGTGACT
Kitl reverse	TGGAAGATTTGCCACCAGTTT
Igf1 forward	TGGATGCTCTTCAGTTCGTG
Igf1 reverse	CACAATGCCTGTCTGAGGTG
Dhh forward	CTTGGCACTCTTGGCACTATC
Dhh reverse	GACCCCCTTGTTACCCTCC
Gapdh forward	AGGTCGGTGTGAACGGATTTG
Gapdh reverse	TGTAGACCATGTAGTTGAGGTCA

References

1. Jameson, S.A. *et al.* Temporal transcriptional profiling of somatic and germ cells reveals biased lineage priming of sexual fate in the fetal mouse gonad. *PLoS Genet* **8**, e1002575 (2012).

2. Kitamura, K. *et al.* Mutation of ARX causes abnormal development of forebrain and testes in mice and X-linked lissencephaly with abnormal genitalia in humans. *Nat Genet* **32**, 359-369 (2002).

3. Shima, Y. *et al.* Contribution of Leydig and Sertoli cells to testosterone production in mouse fetal testes. *Mol Endocrinol* **27**, 63-73 (2013).
