# Supplementary material for: A perivascular niche for multipotent progenitors in the fetal testis
Source: Nat Commun. 2018 Oct 30;9:4519. doi: 10.1038/s41467-018-06996-3 (PMC6207726; doi:10.1038/s41467-018-06996-3)
Supplement: Supplementary file 3 — Reporting Summary [file 41467_2018_6996_MOESM3_ESM.pdf]

nature research

Corresponding author(s): Tony DeFalco

Reporting Summary

Nature Research wishes to improve the reproducibility of the work that we publish. This form provides structure for consistency and transparency in reporting. For further information on Nature Research policies, see Authors & Referees and the Editorial Policy Checklist.

Statistical parameters

When statistical analyses are reported, confirm that the following items are present in the relevant location (e.g. figure legend, table legend, main text, or Methods section).

n/a	Confirmed	
☐	☒	The exact sample size (n) for each experimental group/condition, given as a discrete number and unit of measurement
☐	☒	An indication of whether measurements were taken from distinct samples or whether the same sample was measured repeatedly
☐	☒	The statistical test(s) used AND whether they are one- or two-sided<br>*Only common tests should be described solely by name; describe more complex techniques in the Methods section.*
☒	☐	A description of all covariates tested
☒	☐	A description of any assumptions or corrections, such as tests of normality and adjustment for multiple comparisons
☐	☒	A full description of the statistics including central tendency (e.g. means) or other basic estimates (e.g. regression coefficient) AND variation (e.g. standard deviation) or associated estimates of uncertainty (e.g. confidence intervals)
☐	☒	For null hypothesis testing, the test statistic (e.g. F, t, r) with confidence intervals, effect sizes, degrees of freedom and P value noted<br>*Give P values as exact values whenever suitable.*
☒	☐	For Bayesian analysis, information on the choice of priors and Markov chain Monte Carlo settings
☒	☐	For hierarchical and complex designs, identification of the appropriate level for tests and full reporting of outcomes
☒	☐	Estimates of effect sizes (e.g. Cohen's d, Pearson's r), indicating how they were calculated
☐	☒	Clearly defined error bars<br>*State explicitly what error bars represent (e.g. SD, SE, CI)*

Our web collection on statistics for biologists may be useful.

Software and code

Policy information about availability of computer code

Data collection	No software was used to collect data in this study.
Data analysis	Basic statistical analyses for qPCR assays were performed in Microsoft Excel.

For manuscripts utilizing custom algorithms or software that are central to the research but not yet described in published literature, software must be made available to editors/reviewers upon request. We strongly encourage code deposition in a community repository (e.g. GitHub). See the Nature Research guidelines for submitting code & software for further information.

Data

Policy information about availability of data

All manuscripts must include a data availability statement. This statement should provide the following information, where applicable:

- Accession codes, unique identifiers, or web links for publicly available datasets
- A list of figures that have associated raw data
- A description of any restrictions on data availability

The data that support the findings of this study are available from the authors on reasonable request.

Field-specific reporting

Please select the best fit for your research. If you are not sure, read the appropriate sections before making your selection.

☒ Life sciences ☐ Behavioural & social sciences

For a reference copy of the document with all sections, see nature.com/authors/policies/ReportingSummary-flat.pdf

Life sciences

Study design

All studies must disclose on these points even when the disclosure is negative.

Sample size	No power analyses were performed to determine sample size. A standard n of 3 or greater was sufficient, as each biological replicate had many data points or multiple individual samples within it to perform statistical analyses (for example, looking at number of Leydig cells within each testis for immunofluorescence or performing qPCR analyses in which each independent litter had multiple individual male embryos).
Data exclusions	No data were excluded.
Replication	Experiments were reproduced reliably. The major finding, i.e., increased Leydig cell number after vascular depletion, was validated in multiple, different experimental settings, such as in CD-1 (wild-type), Tomato lineage tracing, and Venus Notch reporter assays. Additionally, findings were verified using independent experimental techniques, such as qPCR and immunofluorescence, when possible. Finally, all assays were repeated in at least 3 biologically independent experiments (often with multiple samples in each biologically independent group).
Randomization	For samples used in organ culture, gonads were randomly chosen for vehicle or experimental treatment (one gonad per condition). Otherwise, samples were grouped by genotype or treatment condition.
Blinding	No specific methods were used for blinding, although virtually all experiments were performed by both authors to virtually identical results.

Materials & experimental systems

Policy information about availability of materials

n/a	Involved in the study
☐	☒ Unique materials
☐	☒ Antibodies
☒	☐ Eukaryotic cell lines
☐	☒ Research animals
☒	☐ Human research participants

Unique materials

Obtaining unique materials	We used anti-ARX antibody (described in Kitamura et al., 2002 Nat Genet), which was provided by Ken-ichirou Morohashi after obtaining permission from Kunio Kitamura; we also used anti-HSD17B3 antibody (described in Shima et al., 2013 Mol Endocrinol), which was provided by Yuichi Shima. These reagents are readily available from the original authors upon request.

Antibodies

Antibodies used

Rabbit anti-SOX9 EMD Millipore #AB5535
Rat anti-PECAM1 BD Biosciences #553370
Rat anti-CDH5 BD Biosciences #550548
Rabbit anti-Nestin Biolegend #PRB-315C
Chicken anti-Nestin Neuromics #CH23001
Goat anti-PECAM1 R&D Systems #AF3628
Goat anti-CYP17A1 Santa Cruz #sc-46081
Goat anti-HSD3B1 Santa Cruz #sc-30820
Rabbit anti-HSD3B1 Cosmo Bio #KAL-KO607
Rabbit anti-ACTA2 Abcam #ab5694
Rabbit anti-NG2 EMD Millipore #AB5320
Rabbit anti-MKI67 (Ki-67) Thermo Fisher Scientific #RM-9106-S
Rat anti-phospho Histone H3 (Ser10) EMD Millipore #MABE939
Rabbit anti-HIF1A Abcam #ab179483
Rabbit anti-cleaved Caspase 3 (Asp175) Cell Signaling #9661S
Chicken anti-GFP Aves #GFP-1020
Rabbit anti-ARX K. Morohashi; Kitamura et al. Nat Genet 2002

Mouse anti-NR2F2 Perseus Proteomics #PP-H7147-00
Rat anti-NR5A1 (SF1) Cosmo Bio #KAL-KO610
Rabbit anti-RFP (tdTomato) Rockland #600-401-379
Goat anti-DLL4 R&D Systems #AF1389
Rabbit anti-NOTCH2 Cell Signaling #5732S
Mouse anti-WT1 (F-6) Santa Cruz #sc-7385
Rat anti-HSD17B3 Y. Shima; Shima et al. Mol Endocrinol 2013

Validation	Commercially available antibodies have provided validation statements on their respective websites. We have performed our own validation and control experiments to ensure specificity of antibodies for immunofluorescence, such as: omitting primary antibody; staining tissues known to lack antigen (either knockout tissue or not containing cell/protein of interest); and co-staining with other antibodies to assess cell-type specificity.

Research animals

Policy information about studies involving animals; ARRIVE guidelines recommended for reporting animal research

Animals/animal-derived materials	We have used mouse (Mus musculus) of various strains, such as CD-1 (outbred), C57BL/6J (B6), transgenic, and/or mixed genetic backgrounds to obtain testis (male) samples between stages embryonic (E) 11.5-E18.5 for fetal studies and P60 for adult studies. Specific mouse strains used were: CD-1; C57BL/6J; B6.Cg-Gt(ROSA)26Sortm14(CAG-tdTomato)Hze/J; Tg(Nes-cre)1Kln/J; Tg(Nes-cre/ERT2,-ALPP)1Sbk; Tg(Cp-HIST1H2BB/Venus)47Hadj/J; Notch2tm2(cre)Rko; and Gt(ROSA)26Sortm3(CAG-EYFP)Hze/J. Mice were housed in accordance with National Institutes of Health guidelines, and experimental protocols were approved by the Institutional Animal Care and Use Committee (IACUC) of Cincinnati Children's Hospital Medical Center (animal experimental protocol numbers IACUC2013-0241 and IACUC2018-0027).

Method-specific reporting

n/a	Involved in the study
☒	☐ ChIP-seq
☒	☐ Flow cytometry
☒	☐ Magnetic resonance imaging
